# Pituitary society expert Delphi consensus: operative workflow in endoscopic transsphenoidal pituitary adenoma resection

**DOI:** 10.1007/s11102-021-01162-3

**Published:** 2021-07-06

**Authors:** Hani J. Marcus, Danyal Z. Khan, Anouk Borg, Michael Buchfelder, Justin S. Cetas, Justin W. Collins, Neil L. Dorward, Maria Fleseriu, Mark Gurnell, Mohsen Javadpour, Pamela S. Jones, Chan Hee Koh, Hugo Layard Horsfall, Adam N. Mamelak, Pietro Mortini, William Muirhead, Nelson M. Oyesiku, Theodore H. Schwartz, Saurabh Sinha, Danail Stoyanov, Luis V. Syro, Georgios Tsermoulas, Adam Williams, Mark J. Winder, Gabriel Zada, Edward R. Laws

**Affiliations:** 1 Division of Neurosurgery, National Hospital for Neurology and Neurosurgery, London, UK; 2 Wellcome/EPSRC Centre for Interventional and Surgical Sciences, University College London, London, UK; 3 Department of Neurosurgery, John Radcliffe Hospital, Oxford, UK; 4 Department of Neurosurgery, University Hospital Erlangen, Erlangen, Germany; 5 Department of Neurosurgery, Oregon Health & Science University, Portland, USA; 6 Department of Uro-Oncology, University College London Hospitals NHS Foundation Trust, London, UK; 7 Departments of Medicine (Endocrinology), Oregon Health & Science University, Portland, USA; 8 Division of Clinical Endocrinology & NIHR Cambridge Biomedical Research Centre, Cambridge University Hospitals NHS Foundation Trust, Cambridge, UK; 9 Wellcome Trust-MRC Institute of Metabolic Science, University of Cambridge, Cambridge, UK; 10 Department of Neurosurgery, National Neurosurgical Centre, Beaumont Hospital, Dublin, Ireland; 11 Department of Neurosurgery, Massachusetts General Hospital, Harvard Medical School, Boston, USA; 12 Department of Neurosurgery and Pituitary Center, Cedars-Sinai Medical Center, Los Angeles, USA; 13 Department of Neurosurgery, San Raffaele University Health Institute Milan, Milan, Italy; 14 Department of Neurosurgery, University of North Carolina at Chapel Hill, Chapel Hill, North Carolina USA; 15 Department of Medicine (Endocrinology), University of North Carolina at Chapel Hill, Chapel Hill, North Carolina USA; 16 Department of Neurosurgery, Weill Medical College of Cornell University, New York, USA; 17 Department of Neurosurgery, Royal Hallamshire Hospital & Sheffield Children’s Hospital, Sheffield, UK; 18 Department of Neurosurgery, Hospital Pablo Tobon Uribe and Clinica Medellin—Grupo Quirónsalud, Medellin, Colombia; 19 Department of Neurosurgery, Queen Elizabeth Hospital Birmingham, Birmingham, UK; 20 Institute of Metabolism and Systems Research, University of Birmingham, Birmingham, UK; 21 Department of Neurosurgery, Southmead Hospital Bristol, Bristol, UK; 22 Department of Neurosurgery, St Vincent’s Public and Private Hospitals, Sydney, Australia; 23 Department of Neurosurgery, University of Southern California, Los Angeles, California USA; 24 Department of Neurosurgery, Brigham and Women’s Hospital, BTM 4, 60 Fenwood Road, Boston, USA

**Keywords:** Endoscopic transsphenoidal surgery, Endoscopic endonasal, Skull base surgery, Pituitary adenoma, Pituitary, Consensus, Delphi

## Abstract

**Purpose:**

Surgical workflow analysis seeks to systematically break down operations into hierarchal components. It facilitates education, training, and understanding of surgical variations. There are known educational demands and variations in surgical practice in endoscopic transsphenoidal approaches to pituitary adenomas. Through an iterative consensus process, we generated a surgical workflow reflective of contemporary surgical practice.

**Methods:**

A mixed-methods consensus process composed of a literature review and iterative Delphi surveys was carried out within the Pituitary Society. Each round of the survey was repeated until data saturation and > 90% consensus was reached.

**Results:**

There was a 100% response rate and no attrition across both Delphi rounds. Eighteen international expert panel members participated. An extensive workflow of 4 phases (nasal, sphenoid, sellar and closure) and 40 steps, with associated technical errors and adverse events, were agreed upon by 100% of panel members across rounds. Both core and case-specific or surgeon-specific variations in operative steps were captured.

**Conclusions:**

Through an international expert panel consensus, a workflow for the performance of endoscopic transsphenoidal pituitary adenoma resection has been generated. This workflow captures a wide range of contemporary operative practice. The agreed “core” steps will serve as a foundation for education, training, assessment and technological development (e.g. models and simulators). The “optional” steps highlight areas of heterogeneity of practice that will benefit from further research (e.g. methods of skull base repair). Further adjustments could be made to increase applicability around the world.

## Background

Endonasal transsphenoidal approaches to the skull base are emerging as the first-line approach for resecting the majority of pituitary adenomas which require surgical intervention [1–3]. However, there is variation in the ways in which these operations are performed, largely based on surgeon preference and training, which may result in differing surgical outcomes [4–7]. These operations are technically demanding, relatively low volume, with steep learning curves—culminating in the frequent requirement for dedicated fellowships to achieve procedure-specific competency [8–11].

Surgical workflow analysis seeks to systematically break down surgical procedures into defined tasks and errors [12, 13]. In this hierarchical process, *procedures* are broken down into *phases* which contain a series of *steps*, generating a dedicated workflow [13]*.* During each step (e.g. suturing), surgical instruments (e.g. forceps) are used to perform manoeuvres (e.g. knot tying) via a series of gestures (e.g. grasping and pulling suture) [14]. Similarly, at each step, there is the potential for technical errors (lapses in surgical technique) and adverse events (an event that may lead to adverse outcomes or postoperative complications) [12].

These workflows may be used for the training (for example, creation of simulations), objective assessment of procedure-specific surgical skills and evaluation of novel surgical technologies or techniques [12, 15–17]. By creating a complimentary nurse and anaesthetic workflow analysis, operating room efficiency may be improved by orchestrating the surgical team [15]. The principal limitation to workflow analysis is the labelling and segmentation of operations into constituent phases, steps and errors, however this process can be automated (or semi-automated) using machine learning techniques [18–20]. The effectiveness of such automation is dependent on the generation of a comprehensive and exhaustive workflow to train deep neural networks to recognise the phases, steps, instruments and errors of an operation.

Consensus processes involving subject experts have been used in order to generate a comprehensive and standardised workflow for named operations [15, 21, 22]. The Delphi technique allows for the generation of group consensus through iterative surveys, interspersed with feedback [23]. Questions nested within surveys can be qualitative or quantitative (often using ordinal scales). If quantitative metrics are used, simplified scales (e.g. 3-point) may translate more clearly into clinical practice with greater test–retest reliability [24]. With an engaged group of experts and the use of digital technologies, the process can be achieved in an accelerated fashion (a matter of weeks) [25]. The management of pituitary adenomas has benefitted from consensus statements, with groups such as the Pituitary Society producing a number of guidelines through its multidisciplinary specialist network [26–32]. However, there is no consensus on the operative workflow for endonasal transsphenoidal approaches (TSA) to pituitary adenomas.

We, therefore, sought to generate a surgical workflow for endoscopic TSA resection of pituitary adenomas, via an expert consensus process nested within the Pituitary Society.

## Methods

### Overview

**Fig. 1:**
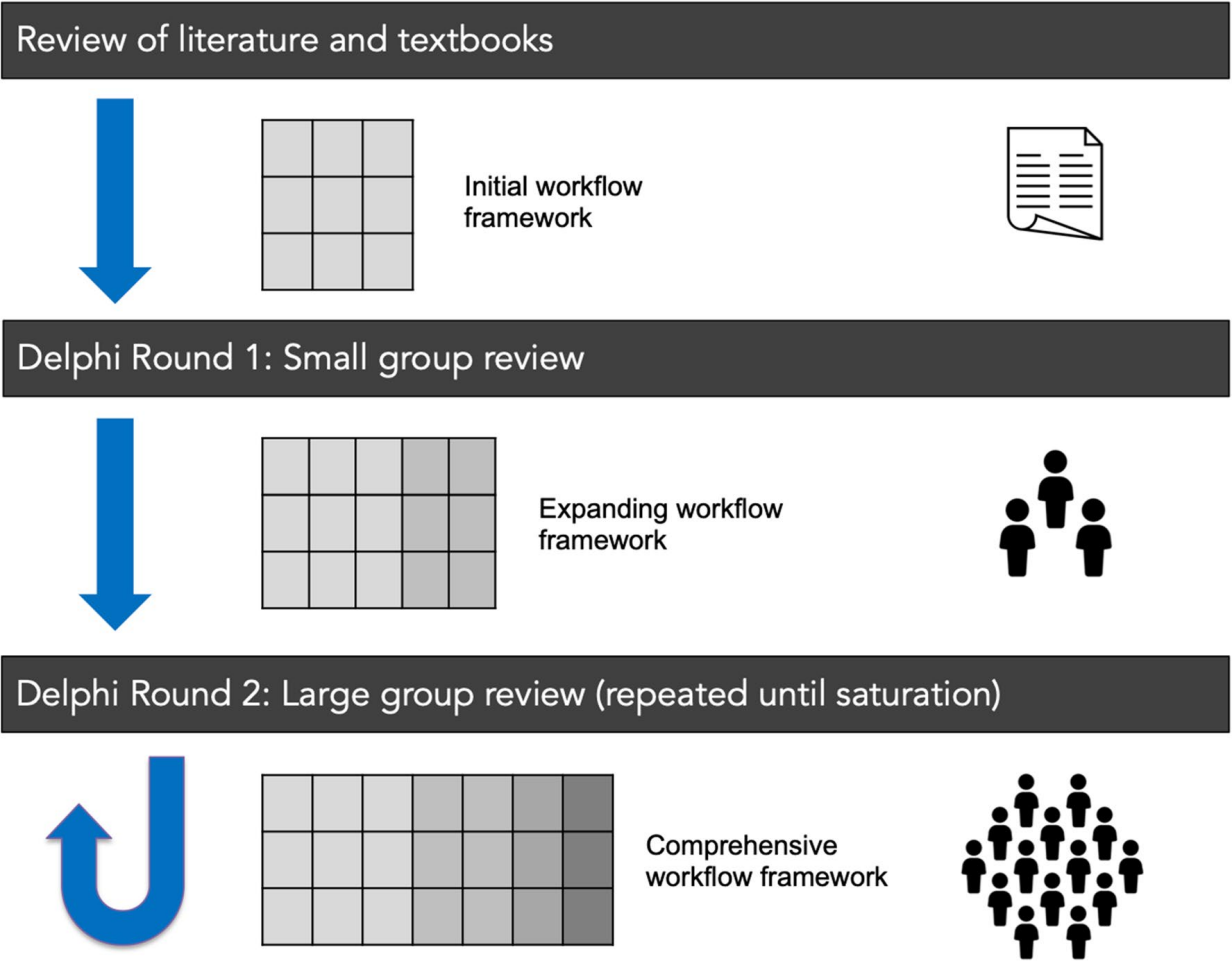
Schematic diagram of Delphi process – highlighting the generation of a surgical workflow through iterative consensus from Pituitary Society expert members

This process aimed to generate a surgical workflow that captured the range of ways the operation is performed in contemporary practice. The aim of the process was not to decide on the optimal set of surgical phases, steps or instruments—this will be explored in subsequent studies. In order to create this exhaustive workflow, expert input was derived through an iterative, mixed-methods consensus process (Fig. 1). The components of the workflow analysis and associated definitions are listed in Table 1 [13, 33]. The beginning of the operation was taken at entry of the endoscope endonasally with the use of surgical instruments, reflecting the American College of Surgeons definition of surgery—“structurally altering the human body by the incision or destruction of tissues” [34].

**Table 1:** Definitions of operative workflow terminology per domain

Domain	Definition	Example
*Phase*	A major event occurring during a surgical procedure, composed of several steps [13]	Nasal phase (endonasal pituitary surgery)—encompassing the beginning of surgery until entry into the sphenoid sinus
*Step*	A sequence of activities used to achieve a surgical objective [13]	Displacement of middle turbinate (endonasal pituitary surgery)
*Instrument*	A tool or device for performing specific actions (such as cutting, dissecting, grasping, holding, retracting, or suturing) during a surgical step	Kerrison Rongeur
*Technical error*	Lapses in operative technique whilst performing a surgical step [33]	Drilling the sella too far laterally (endonasal pituitary surgery)
*Adverse event*	An intraoperative event which is a result of a technical error and has the potential to lead to a post-operative adverse outcome/complication [33]	Carotid artery injury—as a result of drilling the sella too far laterally (endonasal pituitary surgery)

### Modified Delphi process & sampling

#### Literature review

The process (Fig. 1) began with a brief literature review of neurosurgical textbooks and articles (PubMed or EMBASE). Keywords “endoscopic transsphenoidal”, “pituitary adenoma” and “operative technique” were used. From the relevant resources found, an initial operative workflow was generated [5–7, 35, 36].

#### Consensus round 1

The initial, literature-based workflow was discussed with a small group (n = 7) of experts—UK and Ireland based consultant neurosurgeon members of the Pituitary Society. Each expert reviewed the workflow individually—via computerised document (Microsoft Word, Version 16.4, Microsoft, Washington, USA)—with the definitions of phases, steps, instruments, technical errors and adverse events as above. Each expert was asked a series of questions (via e-mail), seeking to assess the completeness and accuracy of the workflow (“Appendix A” section). Any additional suggestions were reviewed and added to the workflow matrix if (i) in-scope, (ii) not duplicate. According to the Delphi technique, circulation and iterative revision of the workflow was repeated until data saturation was achieved, that is, all experts were satisfied that the workflow was complete and accurate. Resultantly, round 1 was repeated three times, occurring over 12 weeks (October 2020–Jan 2021).

#### Consensus round 2

The refined workflow was then sent to a larger group (n = 11)—international members of the Pituitary Society that are recognised experts in the field and nominated by the Physician Education Committee. Again, individuals were asked to assess the workflow (“Appendix A” section), and expand the defined domains (steps, instruments, technical errors and adverse events) to cover possible global variations in practice. As in Round 1, any additional suggestions were reviewed and added to the workflow matrix if (i) in-scope, (ii) not duplicate. This round was completed until (i) all experts agreed that the workflow captures the operative practice they have observed and (ii) there were no additional suggestions for the workflow from the participant group. Round 2 was repeated twice, occurring over 8 weeks (January 2021–March 2021).

### Administration

Invitations to participate in the Delphi process were via direct email only. Workflow documents were presented using Microsoft Word (Version 16.4, Microsoft, Washington, USA) in both rounds and supported by Google Forms (Google LLC, California, USA) in Round 2.

### Data collection and analysis

Participant demographics collected included training grade and country of practice. The collected data regarding the surgical workflow were quantitative (whether participants agree it is complete and accurate) and qualitative (additional suggestions or comments). Summary statistics (e.g. frequencies) were generated for participants demographics. Content analysis was used to analyse free-text responses—to remove out-of-scope suggestions, group similar suggestions together and compare them to existing data points in the workflow. Data analysis and workflow updates were performed in duplicate by two independent analysers (HJM, DZK).

### Ethics

No identifiable data were collected about participants in the Delphi process. This study was independent of national health services and did not require ethical approval (interrogated via online Health Research Authority decision tool—“Appendix B” section) [37].

## Results

### General

There was a 100% response rate and no attrition across both Delphi rounds. Across both rounds, 18 panel members participated, representing seven countries: United Kingdom (n = 6), United States of America (n = 7), Australia (n = 1), Colombia (n = 1), Germany (n = 1), Italy (n = 1) and Republic of Ireland (n = 1).

### Final surgical workflow

Four distinct operative phases were delineated on discussion—nasal, sphenoid, sellar and closure. The component steps within each phase were defined as core (necessary) or optional (case and/or surgeon dependent) and were agreed upon by 100% of panel members across rounds. Pre-operative set-up and post-operative protocols were judged as important but not included as per the defined study scope.

#### Nasal phase

This phase was composed of 10 steps (4 core, 6 optional), from the identification of pertinent nasal anatomy until entry into the sphenoid sinus (Table 2). Amongst our panel, this phase was performed both with otorhinolaryngologists or by neurosurgeons alone.

**Table 2:** The nasal phase with constituent steps, errors and adverse events

Steps		Instruments	Technical error	Adverse event
Core	**Identification of choana, septum, midline, turbinates, anatomic variations**	Suction (to remove mucous)	• Failure to identify correct anatomy	• Failure to progress through or complete steps and increased operative time
Core	**Lateral displacement of middle turbinate and superior turbinate**	Freer elevator	• Laceration of mucosa • Excessive force in bony manipulation (inadvertent entry to maxillary sinus, orbital fracture, cribriform fracture, optic foramen fracture extension)	• Uncontrolled bleeding and epistaxis • Orbital haematoma • Optic nerve injury, other neurovascular injury • CSF leak
Optional	**Turbinectomy (complete or partial)**	Micro-debrider, turbinectomy scissors, endoscopic scissors, thru cut forceps, co-ablation, Colorado needle, needle & piston syringe (adrenaline)	• Failure to protect vasculature (excessive mucosal resection)	• Turbinate artery injury, uncontrolled bleeding, epistaxis
Core	**Identification of sphenoid ostium and sphenoethmoidal recess**	Spatula, Freer elevator, Howarth elevator	• Failure to identify correct anatomy	• Failure to progress through or complete steps and increased operative time
Optional	**Sphenoid ostium coagulation**	Monopolar cautery, suction bipolar, co-ablation		
Optional	**Septal mucosal incision (for “rescue” flap) or full pedicled vascular flap harvest**	Telescopic knife, grasper, Colorado needle, Cottle elevator	• Failure to protect vasculature	• Sphenopalatine or septal artery injury, uncontrolled bleeding • Non-vascularized pedicle
			• Excessively deep incision	• Septal perforation
			• Failure to protect olfactory mucosa	• Hyposmia or anosmia
			• Failure to identify subperiosteal or subperichondrial plane	• Inadequate nasoseptal flap
Core	**Anterior sphenoidotomy**	Kerrison punch, Stammberger punch, high-speed drill, microdebrider	• Failure to protect vasculature (excessive mucosal resection)	• Sphenopalatine artery injury, uncontrolled bleeding, epistaxis • Nasoseptal flap ischaemia (if used)
			• Excessive or mispositioned bony resection (e.g. accessing anterior cranial fossa)	• CSF leak • Carotid injury, other neurovascular injury (e.g. olfactory nerve)
			• Inadequate resection resulting in limited surgical access	• Failure to progress through or complete steps and increased operative time
Optional	**Posterior septectomy**	Cottle elevator (to protect mucosa), microdebrider, Blakesley forceps, Kerrison rongeur, backbiter rongeur, pituitary rongeur, Jansen-Middleton rongeur, high-speed drill, Tilley Henckel forceps, Co-ablation	• Failure to protect vasculature (excessive mucosal resection)	• Sphenopalatine or septal artery injury, uncontrolled bleeding, epistaxis • Nasoseptal flap ischaemia (if used)
			• Excessive septectomy	• Septal perforation • Saddle deformity of the nose
Optional	**Lateral displacement of septum (mononostril approach)**	Kerrison rongeur, Freer elevator	• Excessive force in bony manipulation causing fracture extension	• CSF leak • Neurovascular injury (e.g. olfactory nerves at anterior skull base)
Optional	**Septoplasty (in cases of significant septal deviation)**	Freer elevator, finger	• Excessive force in septal manipulation	• Septal arteries, uncontrolled bleeding, epistaxis • Septal perforation • Saddle deformity
			• Damage to nasal olfactory mucosa	• Hyposmia or anosmia

#### Sphenoid phase

This phase was the shortest in terms of the number of steps, composed of 4 steps (3 core, 1 optional) as detailed in Table 3.

**Table 3:** The sphenoid phase with constituent steps, errors and adverse events

Steps		Instruments	Technical error	Adverse event
Core	**Identification of midline, pneumatization of sphenoid and anatomical variants**	Suction (to remove mucous and blood)	• Failure to identify correct anatomy	• Failure to progress through or complete steps and increased operative time
Core	**Removal or reflection of sphenoid mucosa (partial or total)**	Angel James forceps, grasper, Tilley Henckel forceps, Blakesley punch, microdebrider	• Failure to identify sphenoethmoidal air cell (aka Onodi air cells)	• Optic nerve injury • Carotid injury • Arachnoid tear, CSF leak
Core	**Removal of sinus septations**	Blakesley punch, forward punch, pituitary forceps, Tilley Henckel forceps, Kerrison ronguer, high-speed drill	• Excessive force in bony manipulation	• Skull base fractures
			• Failure to identify sphenoethmoidal air cell (aka Onodi air cells)	• Optic nerve injury • Carotid injury • Arachnoid tear, CSF leak
Optional	**Sinus irrigation**	Large bulb syringe (saline), large piston syringe (saline)		

#### Sellar phase

The sellar phase was composed of 12 steps (7 core, 5 optional) representing entry into the intracranial space and tumour (macroadenoma or microadenoma) resection (Table 4).

**Table 4:** The sellar phase with constituent steps, errors and adverse events

Steps		Instruments	Technical error	Adverse event
Core	**Confirmation of adequate exposure and identification of pertinent landmarks (midline, sellar protuberance, clival recess, tuberculum sellae, optic groove, carotid groove, optic-carotid recess)**	Neuro-navigation	• Failure to identify critical anatomy and exposure adequacy	• Failure to progress through or complete steps and increased operative time • Subsequent neurovascular injury, CSF leak
Core	**Sellotomy**	Nerve hook, chisel, dissector, Kerrison rongeur, Stammberger punch, high-speed drill, Cottle elevator	• Opening too far laterally or over aberrant anatomy	• Carotid injury • Optic nerve injury • Major cavernous sinus injury
			• Inadvertent durotomy	• Carotid injury • Optic nerve injury • Major cavernous sinus injury
			• Excessive or mispositioned bony resection (e.g. accessing anterior cranial fossa)	• CSF leak, other neurovascular injury
			• Inadequate resection resulting in limited surgical access	• Failure to progress through or complete steps and increased operative time
Optional	**Extended skull base resection (e.g. tuberculum sellae resection for large suprasellar component or constricted diaphragm sellae)**	High-speed drill, Kerrison rongeur, Stammberger punch, back-biting rongeur, angled endoscope	• Excessive or mispositioned resection or resection over aberrant anatomy	• Optic nerve injury • Carotid injury • Olfactory nerve injury • CSF leak
			• Inadequate resection resulting in limited surgical access	• Failure to progress through or complete steps and increased operative time
Core	**Confirmation of adequate exposure and identifications of sella limits and neurovascular landmarks (e.g. optic nerves, carotid arteries) with or without adjuncts (micro doppler or neuronavigation)**	Micro Doppler probe, neuro-navigation	• Failure to identify critical anatomy and exposure adequacy	• Failure to progress through or complete steps and increased operative time • Subsequent neurovascular injury, CSF leak
Core	**Durotomy**	Bipolar forceps, Telescopic or retractable knife, endoscopic scissor, sickle knife, bipolar forceps	• Excessive durotomy or over aberrant anatomy	• Carotid injury • Optic nerve injury • Major cavernous sinus injury • Arachnoid tear, CSF leak
			• Inadequate durotomy resulting in limited surgical access	• Failure to progress through or complete steps and increased operative time
Core	**Microadenoma: intracapsular piecemeal or extracapsular en-bloc resection or hemi-hypophysectomy**	Ring curette, suction, microdissector, 11-blade scalpel, saline irrigation	• Excessive pulling on lateral component of the tumour (e.g. causing avulsion of feeding vessel)	• Carotid injury • Major cavernous sinus haemorrhage
			• Direct trauma to surrounding neurovascular structures	• Carotid injury • Major cavernous sinus haemorrhage
			• Excessive traction on diaphragm	• Arachnoid tear, CSF leak
			• Failure to recognise normal gland	• Injury or inadvertent removal of normal gland or stalk
Core	**Macroadenoma: piecemeal resection (usually inferior first, then lateral and then superior)**	Ring curette, suction, small-cup forceps, pituitary rongeurs, Cavitron Ultrasonic Surgical Aspirator (CUSA), Sonopet, saline irrigation	• Excessive pulling on lateral component of the tumour (e.g. causing avulsion of feeding vessel)	• Carotid injury • Major cavernous sinus haemorrhage
			• Direct trauma to surrounding structures or supplying vessels	• Optic nerve injury • Hypothalamic Injury • Basilar artery injury, Carotid artery injury, cerebral (e.g. anterior) artery injury
			• Excessive traction on diaphragm	• Arachnoid tear, CSF leak
			• Premature descent of the diaphragm	• Failure of sufficient tumour resection
			• Failure to recognise normal gland	• Injury or inadvertent removal of normal gland or stalk
Optional	**Cavernous sinus opening**	Blunt-tip angled knife, endoscopic scissors, suction, micro Doppler probe, electro-stimulator probe (for intraoperative nerve monitoring)	• Direct trauma to surrounding structures or supplying vessels	• Carotid injury • Major cavernous sinus haemorrhage • Optic nerve injury • Abducens, trochlear, oculomotor, trigeminal (V1) nerve injury
			• Overpacking of haemostatic materials	• Neurovascular compression
Optional	**Opening of diaphragm**	Suction, microdissector, spatula, bipolar forceps, endoscopic scissors, telescopic knife	• Failure to recognise normal gland or pituitary stalk	• Injury or inadvertent removal of normal gland or stalk
			• Inadequate coagulation of intercavernous sinus	• Major intercavernous sinus haemorrhage
Optional	**Intrathecal saline or air via lumbar drain (if in-situ) to facilitate resection or diaphragm descent**	Saline aliquots	• Instilled volume too small • Instilled volume too large	• Failure of tumour descent • Elevated Intracranial pressure
Optional	**Jugular venous compression or valsalva to facilitate resection or diaphragm descent**		• Insufficient compression or valsalva • Excessive or prolonged compression or valsalva	• Failure of tumour descent • Elevated Intracranial pressure
Core	**Confirmation of adequate resection**	Angled endoscope (0, 30, 45 or 70 degree), neuro-navigation, intra-op MRI	• Failure to identify residual tumour	• Incomplete tumour resection

#### Closure phase

The closure phase was composed of 14 steps (3 core, 11 optional), consisting of haemostasis and repair of the skull base (when appropriate) (Table 5). This phase had the largest number of optional steps, reflecting the acknowledged heterogeneity in the various methods of skull base repair that may be used.

**Table 5:** The closure phase with constituent steps, errors and adverse events

Steps		Instruments	Technical error	Adverse event
Core	**Haemostasis**	Bipolar, suction, cottonoid patties, Blakesley forceps, synthetic agents (e.g. Surgicel, Floseal, Surgiflo), warm saline irrigation	• Failure to achieve haemostasis	• Epistaxis, haematoma, compressive optic nerve injury • Displacement of skull base reconstruction materials (resulting in CSF leak)
			• Overpacking of haemostatic materials	• Neurovascular compression (e.g. optic nerve at sellar region or abducens nerve at cavernous region)
Core	**Inspection for occult CSF leak**	Angled endoscope (0, 30, 45 or 70 degree), suction, intrathecal fluorescein, ventilator (for valsalva), saline aliquots (via lumbar drain)	• Failure to identify and repair arachnoid breach	• CSF leak
Optional	**Free graft harvesting**	Thigh or abdomen (scalpel, retractor, scissors, bipolar, forceps, sutures, needle holder)	• Failure to achieve haemostasis	• Uncontrolled bleeding, haematoma
			• Failure to reduce dead space on closure	• Haematoma, seroma
			• Excessively deep or wide incision	• Damage to surrounding structures
		Nasal mucosa or bone (Blakesley forceps, Cottle elevator, telescopic knife, endoscopic scissors, co-ablation, bipolar, Colorado needle)	• Failure to achieve haemostasis	• Uncontrolled bleeding, epistaxis, haematoma
			• Sphenopalatine, middle or inferior turbinate artery injury	• Uncontrolled bleeding, epistaxis, haematoma
			• Excessively deep or wide incision	• Damage to surrounding structures. septal perforation
Optional	**Dural repair or reconstruction**	Synthetic grafts (e.g. Duragen), Autologous grafts (e.g. Fascia Lata), Blakesley forceps, suction, sutures, clips	• Failure to achieve a watertight seal	• CSF leak
Optional	**Sellar packing (fat, synthetic material)**	Suction, Blakesley forceps, Tilley dressings forceps	• Overpacking in the fossa • Underpacking	• Optic nerve compression • Optic chiasmatic collapse
			• Failure to achieve a watertight seal	• CSF leak
Optional	**Vascularised flap *placement*** **(*harvesting* of flap precedes this and may be performed here or in the nasal phase—please see dedicated step in the nasal phase)**	Suction, Freer elevator, Cottle elevator	• Failure to achieve a watertight seal • Sphenopalatine, middle turbinate or inferior turbinate artery injury • Avulsion of flap	• CSF leak • Uncontrolled bleeding, epistaxis, haematoma, vascular flap ischaemia • Vascular flap ischaemia
Optional	**Tissue glues**	Glue applicator	• Failure to achieve watertight seal or maintain repair construct	• CSF leak
Optional	**Placement of supportive rigid buttress**	Suction, Blakesley forceps, Tilley dressings forceps	• Failure to support reparative construct or maintain repair construct	• CSF leak
Optional	**Medialising turbinates**	Freer elevator	• Excessive force exertion • Insufficient force exertion or insufficient displacement	• Avulsion • Sinonasal obstruction
Optional	**Medialising septum**	Freer elevator	• Excessive force exertion • Insufficient force exertion or insufficient displacement	• Septal perforation, septal deformity • Sinonasal obstruction, septal deformity
Core	**Clearance of debris (e.g. at nasopharynx)**	Suction	• Failure to clear debris	• Aspiration
Optional	**Placement of nasal packs (e.g. balloon-based or gauze-based)**	Pituitary rongeurs, cup forceps	• Failure to support reparative construct • Excessive pressure	• CSF leak • Flap ischaemia, optic nerve compression
Optional	**Placement of nasal silastic splints**	Sutures, needle holder, forceps	• Excessive pressure • Insufficient securing	• Septal perforation, flap ischaemia • Migration, nasal obstruction
Optional	**Placement of lumbar drain (may be pre- or post-op)**	Lumbar drain needle, drain tubing, drainage system	• Under-drainage	• CSF leak
			• Over-drainage	• Subdural haematoma
			• Incorrect needle placement	• Neurovascular injury, uncontrolled bleeding
			• Contamination	• Bacterial colonisation or infection
			• Drain tubing secured or connected incorrectly	• Tube dislocation, blockage

## Discussion

### Principal findings

Firstly, a workflow for the performance of endoscopic transsphenoidal pituitary adenoma resection has been generated, using Delphi methodology based on an international expert consensus agreement. The agreed “core” steps can be used for education (e.g. operative video annotation), surgical skills assessment, and the development of models and simulators [13, 19, 22, 38]. Similarly, the agreed “optional” steps highlight areas of heterogeneity of practice that will benefit from further research—most notably in skull base reconstruction (closure phase) and surgical exposure (nasal, sphenoid, sellar phases) [2, 3, 5, 7, 39]. This workflow also captures the instruments, errors and adverse events for each step and is the first of its kind in neurosurgery.

Furthermore, ensuring that the workflow captured a breadth of operative practice, in a structured fashion with consistent terminology, was a challenge and required multiple iterations across multiple rounds. For example, the presence of “optional” steps reflects differences between the practice of individual surgeons (e.g. choice of reparative material) and adaptation to case-specific factors (e.g. tumour extension) [5, 7, 40]. Resultantly, delineation of whether these steps were core or optional and the content of these steps (particularly instrument use) was an area of the workflow which required significant revisions. Similarly, another area that required significant iterative changes was distinguishing errors from adverse events and complications. Definitions of each of these components were therefore presented repeatedly, throughout each round. Adverse events were linked in line to particular technical errors and were limited to intra-operative consequences (as opposed to post-operative complications which occur later and more likely to be multifactorial) [33]. Many adverse events linked to particular technical errors were related to the damage of distinct anatomical structures (e.g. carotid artery) which often overlapped across adverse events with a step. Driven by consensus, the terminology was often broadened (e.g. “neurovascular injury, e.g. carotid artery injury”) to capture a breadth of events whilst decreasing repetition within steps and improving the readability of the workflow.

### Findings in the context of existing literature

This Delphi consensus methodology has been used in various surgical specialities to generate similar surgical workflows, with demonstrated utility as a method to consolidate complex opinions into practical workflows [15, 17, 21, 22]. For example, a workflow for steps and errors in laparoscopic surgery by Bonrath et al. focussed on the need for standardised steps and errors for education and structured assessment of trainees [33]. Kaijser et al. explored the steps of laparoscopic bypass and sleeve gastrectomy in detail, deconstructing them further into constituent tasks in order to develop advanced simulators and training curricula [21]. Previous studies have tailored the workflow analysis to different levels of learners, for example, Dharamsi et al. highlighted the need and utility of a consensus-driven workflow for bougie-assisted cricothyroidotomy aimed specifically at novices [22]. A more in-depth analysis is occasionally performed to task or gesture level (which together make up a surgical step), and this level of granularity has been achieved through similar Delphi consensus techniques [41]. Notably, the terminology for the operative workflow hierarchy (e.g. phases, steps, tasks, gestures, motions) is not used in a standardised fashion (e.g. often task and step are used interchangeably) and alignment of future studies to a common language will be important as this field expands [13].

There are many applications of surgical workflows—including education and training; surgical assessment; research; and technology development. In relation to education, highlighting the core components of operations is a useful learning resource for training surgeons and has been used to develop educational curricula, courses and simulators [13, 38]. Similarly, these workflows can be used to inform objective assessment instruments specific to particular operations, for example, Knight et al. combined a consensus-driven surgical steps workflow for laparoscopic hysterectomy with an established skills assessment form (Objective structured assessment of technical skill or OSATS) to generate a reliable and specific measure of procedural proficiency [42]. Augmented assessment and training is particularly pertinent in low-volume surgeries, with steep learning curves and a unique set of surgical skills—such as pituitary surgery [8–10]. Resultantly, proficiency in such procedures requires dedicated fellowships and competency-based assessments, with services providing these operations becoming increasingly consolidated into centres of excellence [10, 26]. Operative workflows may facilitate this through standardisation of terminology, providing a platform to build education materials and specific skills assessments, and highlighting acceptable variations in contemporary practice [13].

A complimentary and related process to surgical workflow analysis is the segmentation of operative videos [13]. For example, focussing on laparoscopic colorectal surgery, Dijkstra et al. distilled the key operative steps—intending to use this information to segment operative videos into component steps [15]. These segmented videos are integrated into the intra-operative environment, to guide and assess trainee surgeons in a uniform fashion [15]. Indeed, such segmentation and procedure-specific analysis has been presented in live operations in animals, displaying an ability to improve the efficiency of tasks and reduce operative times [17]. A disadvantage of operative video segmentation is its labour-intensive nature, however, this process can be automated (or semi-automated) using machine learning techniques [18–20]. Indeed, in the context of the COVID-19 pandemic, where operative caseload is reduced (therefore maximising learning from each case is important) and waiting list backlog is at its highest (therefore more efficient surgery is important), these technologies may be particularly useful [43–45].

### Strengths and limitations

There are several limitations to this study that are important to highlight. Whilst the Delphi method is useful for capturing and refining the opinions of various stakeholders, attention to expert panel selection will naturally influence process output [46]. In our study, our expert panel was international and multicentre. As expected, multicentre consensus processes are capable of identifying a broader and more granular workflow than single centre analyses [21, 47]. However, only one (of 18) expert panel members represented a low or middle-income country and thus our results may not reflect a global operative workflow for this procedure. Moreover, rating regarding the utility or rationale for operative steps (particularly optional steps) was not characterised in this study and this is certainly a point for further study. Finally, pre-operative set-up (e.g. nasal preparation and patient positioning) and post-operative strategies (e.g. placement of a nasogastric tube) were excluded for practical and scope purposes, and this again is an area that requires further study to characterise heterogeneity and explore comparative effectiveness.

## Conclusions

Through an international expert panel consensus, a workflow for the performance of endoscopic transsphenoidal pituitary adenoma resection has been generated. This workflow captures a wide range of contemporary operative practice. The agreed “core” steps will serve as a foundation for education, training, assessment and technological development (e.g. models and simulators). The “optional” steps highlight areas of heterogeneity of practice that will benefit from further research (e.g. methods of skull base repair). Further adjustments could be made to increase applicability around the world.

## Data Availability

Available upon reasonable request.

## Appendices

### Appendix A: guidance questions to experts during each consensus round

#### Round 1:

Q1. Do you think the presented workflow framework encapsulates your own operative practice and practice that you have observed?

If answered “No” to Q1:

Q2. Are there any additional operative steps which you feel should be added?

Q3. Are there any instruments used which are not represented in this framework? If so, at which step(s) would they be most appropriately place?

Q4. Are there any technical errors not listed in the framework? If so, at which step(s) would they be most appropriately place?

Q5. Are there any adverse events not listed in the framework? If so, at which step(s) would they be most appropriately place?

#### Round 2

A. Nasal Phase.

A1. Are there any additional operative steps which you feel should be added OR would you change any of the step contents?

A2. If yes, what would you change?

B. Sphenoid Phase.

B1. Are there any additional operative steps which you feel should be added OR would you change any of the step contents?

B2. If yes, what would you change?

C. Sellar Phase.

C1. Are there any additional operative steps which you feel should be added OR would you change any of the step contents?

C2. If yes, what would you change?

D. Closure Phase.

D1. Are there any additional operative steps which you feel should be added OR would you change any of the step contents?

D2. If yes, what would you change?

## Abbreviations

eTSA: Endoscopic Transsphenoidal Approach
UK: United Kingdom
USA: United States of America
COVID-19: Coronavirus Disease 2019
